# Supplementary material for: Association between Individual Norepinephrine Transporter (NET) Availability and Response to Pharmacological Therapy in Adults with Attention-Deficit/Hyperactivity Disorder (ADHD)
Source: Brain Sci. 2022 Jul 22;12(8):965. doi: 10.3390/brainsci12080965 (PMC9331391; doi:10.3390/brainsci12080965)
Supplement: Supplementary file 1 [file brainsci-12-00965-s001.zip › brainsci-1779576-supplementary.pdf]

Supplementary Materials

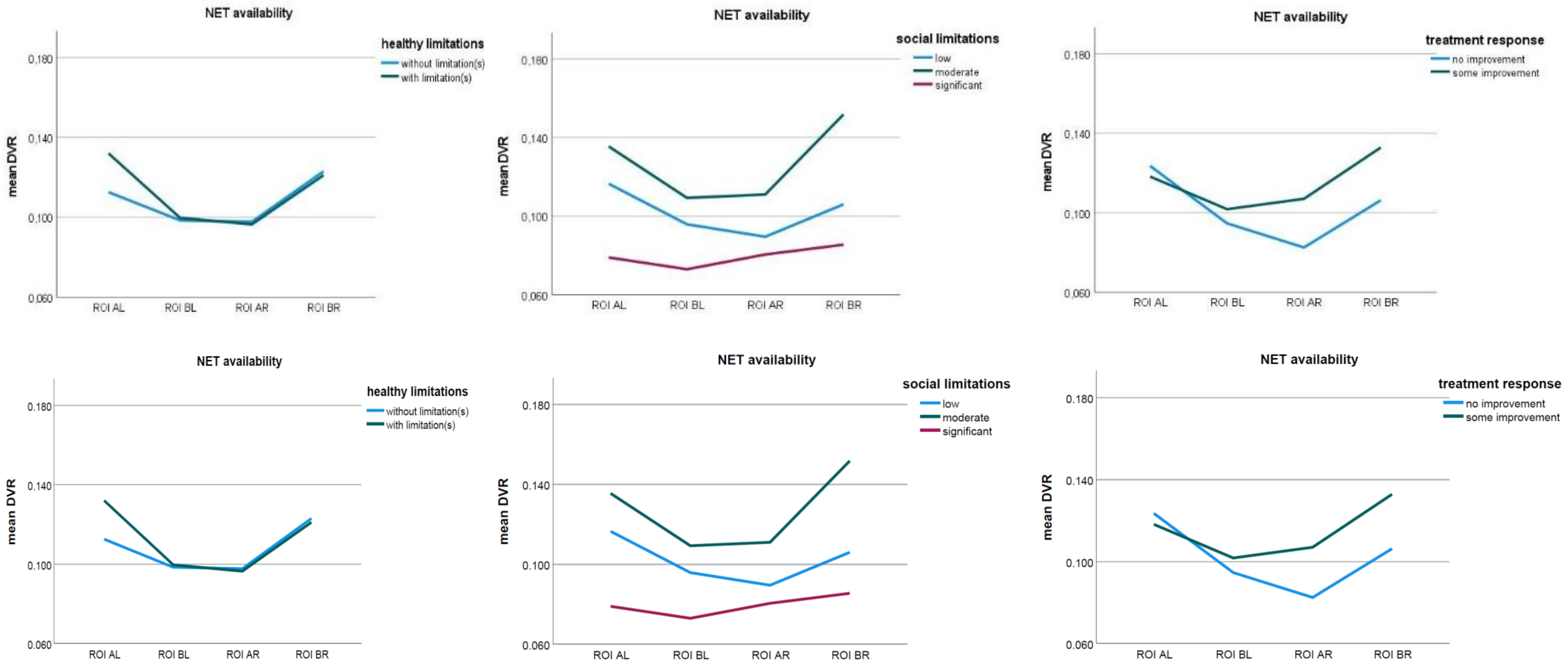

(**a**) (**b**) (**c**)

Figure S1. Mean distributed volume ratios (DVRs) in selected regions of interest (ROIs) for (a) patients with and without diagnosed comorbidities and/or physical disease; (b) patients with different levels of social impairments; (c) patients with and without improvement in CAARS DSM-Global raw scores. Note: AL, attention-related ROI of left hemisphere; BL, behavior-related ROI of left hemisphere; AR, attention-related ROI of right hemisphere; BR, behavior-related ROI of right hemisphere.
